# A descriptive study of routine laboratory testing in intensive care unit in nearly 140,000 patient stays

**DOI:** 10.1038/s41598-022-25961-1

**Published:** 2022-12-13

**Authors:** Jérôme Allyn, Marjolaine Devineau, Matthieu Oliver, Guillaume Descombes, Nicolas Allou, Cyril Ferdynus

**Affiliations:** 1 Intensive Care Unit, Saint-Denis University Hospital, Saint-Denis, Reunion Island, France; 2 Clinical Informatics Department, Saint-Denis University Hospital, Saint-Denis, Reunion Island, France; 3 Biology Laboratory, Saint-Denis University Hospital, Saint-Denis, Reunion Island, France; 4 Methodological Support Unit, Saint-Denis University Hospital, Saint-Denis, Reunion Island, France; 5 INSERM, CIC 1410, 97410 Saint-Pierre, France

**Keywords:** Diagnosis, Health care economics

## Abstract

To describe the relationship between the use of laboratory tests and changes in laboratory parameters in ICU patients is necessary to help optimize routine laboratory testing. A retrospective, descriptive study was conducted on the large eICU-Collaborative Research Database. The relationship between the use of routine laboratory tests (chemistry and blood counts) and changes in ten common laboratory parameters was studied. Factors associated with laboratory tests were identified in a multivariate regression analysis using generalized estimating equation Poisson models. The study included 138,734 patient stays, with an ICU mortality of 8.97%. For all parameters, the proportion of patients with at least one test decreased from day 0 to day 1 and then gradually increased until the end of the ICU stay. Paradoxically, the results of almost all tests moved toward normal values, and the daily variation in the results of almost all tests decreased over time. The presence of an arterial catheter or teaching hospitals were independently associated with an increase in the number of laboratory tests performed. The paradox of routine laboratory testing should be further explored by assessing the factors that drive the decision to perform routine laboratory testing in ICU and the impact of such testing on patient.

## Introduction

The management of patients in intensive care unit (ICU) is a complex process that involves paraclinical examinations, and in particular routine laboratory tests. These tests, which can be performed up to several times a day, every day, are used to establish diagnosis, generally during the initial phase of intensive care hospitalization. They also serve to monitor metabolic disorders, to detect organ failure, and to assess the effectiveness and side effects of treatments throughout the ICU stay.

However, the use of routine laboratory tests in ICU is often inappropriate^1–5^. On the one hand, test overuse can result in blood spoliation and excess cost for health facilities^1,2,6–9^. On the other hand, the underuse of tests can lead to a delay in therapeutic intervention and, consequently, to a loss of chance and a waste of money^1,2^. While overuse is associated with overly permissive medical decision-making, underuse may be the result of overly restrictive criteria^1,3^.

Since doing more with less is a priority for health care systems across the world, the use of routine laboratory tests in ICU must be optimized. To achieve this, there is a need for detailed descriptions of current laboratory testing that draw on massive, recent and representative data on “real life” clinical practice. An assessment of the relationship between laboratory testing and the evolution of laboratory parameters in ICU can also deepen our understanding of the issue. To our knowledge, no such assessment has been conducted to date.

The aim of this retrospective descriptive study was to assess the relationship between the use of routine laboratory tests (chemistry and complete blood count (CBC)) and the evolution of 10 common laboratory parameters in a large sample of nearly 140,000 ICU patient stays.

## Methods

### Study population

The study population was drawn from the eICU Collaborative Research Database (eICU-CRD), a multi-center ICU database containing high granularity data on 200,859 patient ICU stays and 139,367 patients admitted to one of 335 units in 208 hospitals located throughout the US^10^. This deidentified database covers the 2014–2015 period and includes vital sign measurements, care plan documentation, measures of illness severity, information on diagnosis and treatment, etc.

An independent study has confirmed that the data included in the eICU-CRD are complete and reliable^11^. This study received a favorable approval from the ethical commission of the “Société de Réanimation de Langue Française” (reference CE SRLF17-09) and as the re-identification risk associated with this database was certified to meet safe harbor standards by an independent privacy expert, our study was exempt from individual consent. The research reported in this paper adhered to the Helsinki Declaration as revised in 2013, and institutional ethical standards.

### Patient and intensive care unit characteristics

The following patient and ICU characteristics were collected from the eICU-CRD: age, sex, Acute Physiology and Chronic Health Evaluation (APACHE) score, APACHE diagnostic group, need for organ support, use of arterial catheter, length of stay in ICU, length of stay in hospital, death in ICU, hospital teaching status, type of ICU, and number of ICU beds^12,13^.

Patients were removed from the study sample if they met the following exclusion criteria: age under 18 years or over 89 years, APACHE score prediction error, recorded date of ICU discharge preceding the recorded date of ICU admission.

### Laboratory parameters

The analysis focused on 10 common laboratory parameters that were classified into 2 groups. The first group consisted of chemistry parameters: plasma sodium, potassium, chloride, bicarbonate, urea, and creatinine. The second consisted of CBC parameters: white blood cell (WBC) count, red blood cell (RBC) count, hemoglobin levels, and platelet count.

All laboratory parameters were analyzed for the first 8 days of ICU stay, namely from Day-0 (admission) to Day-7, whether venous or arterial in origin, measured on point-of-care testing instruments or core laboratory analyzers.

### Statistical analysis

Categorical variables were expressed as frequencies (percentages) and continuous variables as means (standard deviations) or medians (interquartile ranges).

We chose to exclude laboratory results that were clinically improbable using a simple trimming with a non-parametric method. Thus, we considered as an outlier, a laboratory result that were below the 1 thousandth or above the 999 thousandth of each distribution after checking manually that excluded values were clinically improbable (i.e. measurement errors,..). In addition, we checked that distributions were not too skewed graphically before and after exclusions.

For each laboratory parameter, the proportion of patients who underwent at least 1 test on a single day (i.e. at least one chemistry test regardless of type of analyte on a single day) out of all patients in ICU on that day was evaluated along with test results and daily variations in test results. Daily variations were expressed as percentages of variations for each lab result by calculating the proportion of variation between each day. If more than one lab result was present for a specific day for a patient, we used the median of these results for that day.

A multivariate regression using generalized estimating equations (GEE) Poisson models with log link function was performed to identify the factors independently associated with the number of chemistry and CBC tests performed per patient per day in ICU. The center was included as a clustering factor and an exchangeable correlation matrix was specified. The two-by-two daily variations in laboratory parameters were assessed from this model using contrasts.

An exploratory analysis of the association between ICU mortality and selected variables (sex, age by decade, type of ICU, APACHE diagnostic group, APACHE score) according to hospital teaching status was performed using a logistic regression model. The fit of the final model was assessed using the Hosmer–Lemeshow test.

All analyses were performed at a 2-tailed type I error of 5% using SAS 9.4 (SAS Institute Inc., Cary, NC) and Python 3.8.

### Ethics approval and consent to participate

We received approval from the Institutional Ethics and Research Committee of the Societe de Reanimation de Langue Française (CE-SRLF17-09). The research reported in this paper adhered to the Helsinki Declaration as revised in 2013, and institutional ethical standards.

## Results

### Study population

A total of 62,125 patient stays were excluded: 54,335 due to APACHE score prediction error (as recommended by Pollard et al.^10^)*,* 7,081 patient stays due to age over 89 years, 625 patient stays due to age under 18 years, and 84 patient stays due to a recorded date of ICU discharge preceding the recorded date of admission. Thus, 138,734 patient stays hospitalized in ICU in 2014 and 2015 were included in our analyses^10^. The characteristics of patients and ICUs are shown in Table 1. The comparison of included patient stays with excluded patient stays due to APACHE score prediction error is presented in Supplementary information (Table S1).

**Table 1:** Patient stays and intensive care unit characteristics.

Characteristics^a^	Missing data	Total (n = 138,734)
**Age**, years	0 (0)	62.17 ± 16.48
**Sex**	18 (0.01)	
Female		62,858 (45.31)
Male		75,858 (54.69)
**APACHE score on admission**	0 (0)	55.04 ± 25.52
**APACHE diagnostic group**	0 (0)	
Cardiac arrest		9418 (6.79)
Cardiovascular, acute coronary syndrome		8196 (5.91)
Cardiovascular, congestive heart failure/cardiogenic shock		5656 (4.08)
Cardiovascular, other		3727 (2.69)
Acute renal failure		1933 (1.39)
Respiratory, asthma/emphysema		4178 (3.01)
Respiratory, pneumonia		4746 (3.42)
Respiratory, other		8811 (6.35)
Neurology, cerebrovascular accident		9568 (6.90)
Neurology, coma		2222 (1.60)
Neurology, other		4868 (3.51)
Diabetic ketoacidosis/hyperglycemic hyperosmolar nonketotic coma		4370 (3.15)
Gastrointestinal bleeding		7348 (5.30)
Gastrointestinal obstruction		1211 (0.87)
Chest pain unknown		759 (0.55)
Overdose		4200 (3.03)
Cardiac surgery, valve		2833 (2.04)
Cardiac surgery, coronary artery bypass grafting		5220 (3.76)
Sepsis		18,002 (12.98)
Trauma		5525 (3.98)
Other		25,943 (18.70)
**Catecholamine**	0 (0)	20,547 (14.81)
**Mechanical ventilation**	0 (0)	34,152 (24.62)
**Use of arterial catheter**	0 (0)	38,028 (27.41)
**Hospital teaching status**	0 (0)	
Non-teaching		100,085 (72.14)
Teaching		38,649 (27.86)
**Type of ICU**	0 (0)	
Cardiac ICU		30,885 (22.26)
Medical ICU		12,178 (8.78)
Medical-Surgical ICU		75,698 (54.56)
Neuro-ICU		10,764 (7.76)
Surgical ICU		9209 (6.64)
**Number of hospital beds**	14,680 (10.58)	
< 100		29,272 (23.60)
100 to 249		32,483 (26.18)
250 to 499		7933 (6.39)
≥ 500		54,366 (43.82)
**Length of stay in ICU, days**	0 (0)	3.49 ± 4.20
**Length of stay in hospital, days**	0 (0)	7.44 ± 9.14
**Death in ICU**	0 (0)	12,447 (8.97)

APACHE, Acute Physiology and Chronic Health Evaluation; ICU, Intensive Care Unit.

^a^Results are expressed as n (%) or mean ± standard deviation.

### Number of laboratory tests performed per patient per day in intensive care unit

Number and percentage of laboratory results considered as outliers are presented in Supplementary information (Table S2), according to the methodology chosen the proportion of outliers varied between 0.14 and 0.20%.

The median [25th–75th] time between laboratory tests was 10 [5–23] hours for chemistry tests and 14 [6–24] hours for CBC tests. The mean number of chemistry tests per patient per day varied between 2.67 ± 1.98 on Day-0 and 1.13 ± 1.19 on Day-1, and the mean number of CBC tests per patient per day varied between 2.42 ± 1.81 on Day-0 and 0.95 ± 0.93 on Day-2. Table 2 shows the mean number of chemistry and CBC tests performed per patient per day during the first 8 days of ICU stay.

**Table 2:** Number of chemistry and complete blood count tests performed per patient per day during the first 8 days of intensive care unit stay.

Day	Number of patient stays	Type of test	Mean	Standard Deviation	Median	5th percentile	95th percentile
0	138,734	Chemistry	2.67	1.98	2	1	7
		Complete blood count	2.42	1.81	2	1	6
1	103,604	Chemistry	1.13	1.19	1	0	3
		Complete blood count	0.97	1.01	1	0	3
2	61,623	Chemistry	1.15	1.12	1	0	3
		Complete blood count	0.95	0.93	1	0	3
3	39,478	Chemistry	1.20	1.11	1	0	3
		Complete blood count	0.95	0.88	1	0	2
4	27,382	Chemistry	1.25	1.12	1	0	3
		Complete blood count	0.96	0.86	1	0	2
5	20,134	Chemistry	1.28	1.12	1	0	3
		Complete blood count	0.96	0.84	1	0	2
6	15,381	Chemistry	1.30	1.11	1	0	4
		Complete blood count	0.98	0.82	1	0	2
7	12,169	Chemistry	1.30	1.11	1	0	3
		Complete blood count	0.98	0.81	1	0	2

### Daily variations in the number of laboratory tests performed per patient

A contrast analysis was conducted to measure daily variations in the number of chemistry and CBC tests performed per patient stay. For chemistry parameters, there was a significant decrease in the number of tests performed on Day-2 and Day-3 and a significant increase in the number of tests performed on Day-5, Day-6 compared to the previous day. For CBC parameters, there was a significant decrease in the number of tests performed on Day-2, Day-3, and Day-4 and a significant increase in the number of tests performed on Day-6 compared to the previous day. The results of the contrast analysis are presented in Table 3.

**Table 3:** Daily variations in the number of chemistry and complete blood count tests performed per patient.

Day	Chemistry tests							Complet blood count tests						
	Mean estimation	Mean		Beta	Beta		*p*	Mean estimation	Mean		Beta	Beta		*p*
		Confidence interval			Confidence interval				Confidence interval			Confidence interval		
D2 vs. D1	0.8392	0.8321	0.8463	-0.1753	− 0.1838	− 0.1669	< 0.0001	0.8430	0.8361	0.8500	− 0.1708	− 0.1790	− 0.1626	< 0.0001
D3 vs. D2	0.9453	0.9352	0.9555	− 0.0563	− 0.0670	− 0.0455	< 0.0001	0.9347	0.9250	0.9446	− 0.0675	− 0.0779	− 0.0570	< 0.0001
D4 vs. D3	1.0031	0.9904	1.0160	0.0031	− 0.0096	0.0159	0.6315	0.9828	0.9708	0.9951	− 0.0173	− 0.0297	− 0.0049	0.0061
D5 vs. D4	1.0174	1.0028	1.0322	0.0172	0.0028	0.0317	0.0195	1.0064	0.9925	1.0206	0.0064	− 0.0075	0.0204	0.3671
D6 vs. D5	1.0268	1.0110	1.0428	0.0265	0.0110	0.0420	0.0008	1.0257	1.0109	1.0408	0.0254	0.0109	0.0400	0.0006
D7 vs. D6	1.0114	0.9943	1.0287	0.0113	− 0.0057	0.0283	0.1930	1.0106	0.9943	1.0272	0.0105	− 0.0058	0.0268	0.2051

### Relationship between the use of laboratory tests and the evolution of laboratory parameters

For each laboratory parameter, the proportion of patients who underwent at least 1 test on a single day out of all patients in ICU on that day was evaluated along with test results and daily variations in test results.

**Figure 1:**
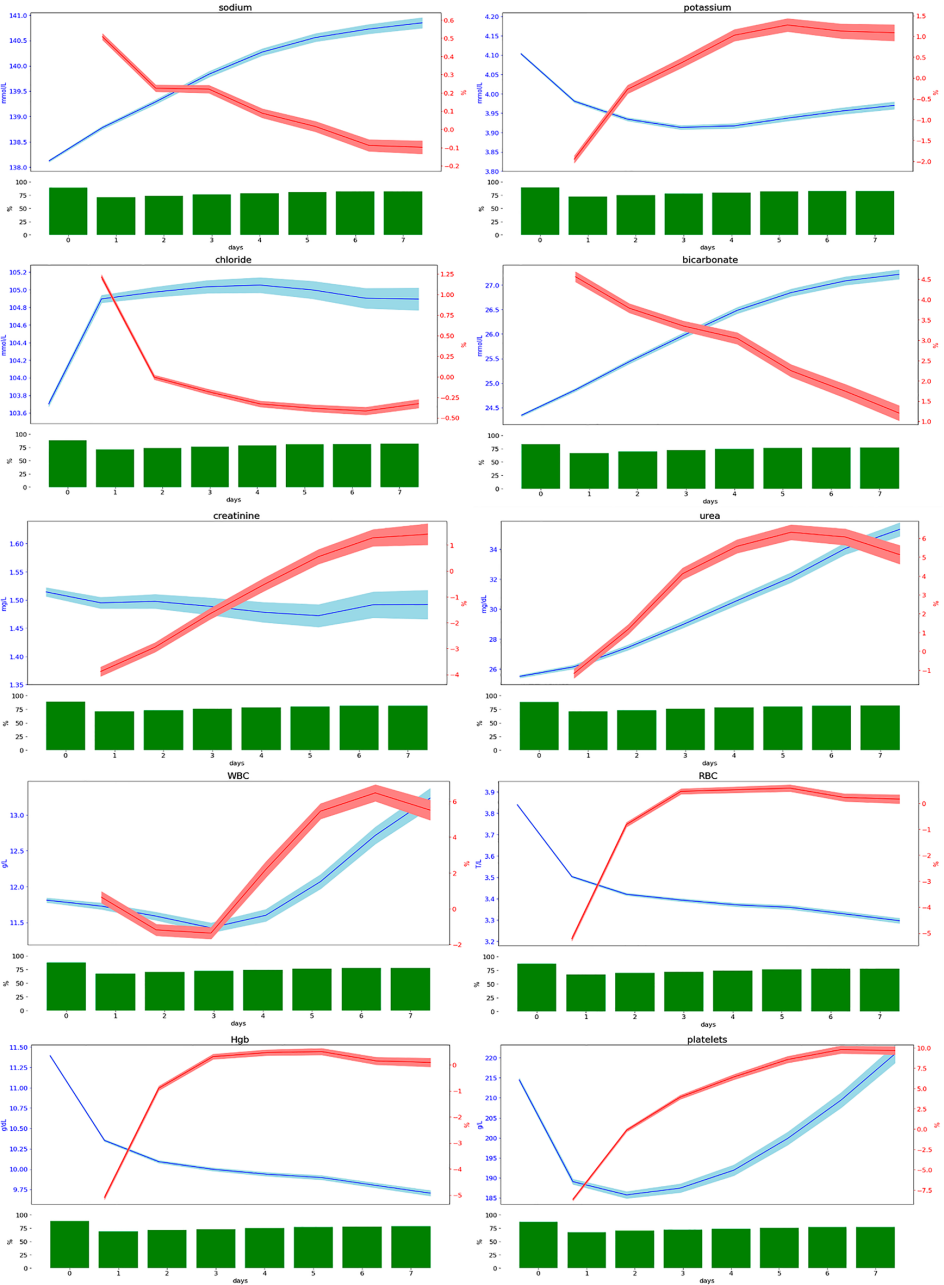
Proportion of patients who underwent at least 1 test on a single day out of all patients in intensive care unit on that day (green histogram), test results (blue curve), and daily variations in test results (red curve). WBC, white blood count; RBC, red blood count: Hgb, hemoglobin.

At Day-0, 90% of patients underwent a least a laboratory test (Fig. 1). This proportion decreased after Day-0, but remained at a high level, 67–70%, at day-1 and 77–82% at day-7 (Fig. 1).

All chemistry parameters evolved towards normal values over time (*i.e.* tend towards the average of the bounds of the norm). The only exception was urea, whose levels increased during the ICU stay. As regards CBC parameters, there was a decrease in hemoglobin levels and RBC count, both of which are markers of anemia.

For all parameters, daily variations in test results decreased over time during the ICU stay, with the exception again of urea. Some laboratory parameters were more finely regulated than others. Thus, daily variations in sodium levels were less than 0.5% compared to daily variations of up to 10% for platelet count.

Figure 1 shows the proportion of patients who underwent at least 1 test on a single day out of all patients in ICU on that day (green histogram) along with test results (blue curve) and daily variations in test results (red curve). The line thickness represents confidence intervals.

### Factors independently associated with the number of laboratory tests performed per patient per day

A multivariate regression analysis using GEE Poisson models was performed to identify factors independently associated with the number of laboratory tests performed per patient per day in ICU. To simplify the analysis, one model was developed for chemistry tests (plasma sodium, potassium, chloride, bicarbonate, urea, and creatinine) and another for CBC tests (WBC count, RBC count, hemoglobin levels, and platelet count).

The following factors were entered in the models: day of hospitalization in ICU, sex, age by decade, use of arterial catheter, type of ICU, APACHE diagnostic group, APACHE score, hospital teaching status. The multivariate regression analysis using GEE Poisson models is summarized in Table 4.

**Table 4:** Multivariate regression using GEE Poisson models.

Variable	Chemistry parameters				Complete blood count parameters			
	Incidence ratio	Confidence interval 95%		*p*	Incidence ratio	Confidence interval 95%		*p*
**Day**								
0	0.0000	0.0000	0.0000		0.0000	0.0000	0.0000	
1	− 0.9243	− 0.9305	− 0.9181	< 0.0001	− 0.9702	− 0.9737	− 0.9616	< 0.0001
2	− 1.0996	− 1.1085	− 1.0908	< 0.0001	− 1.1410	− 1.1474	− 1.1307	< 0.0001
3	− 1.1559	− 1.1670	− 1.1448	< 0.0001	− 1.2084	− 1.2156	− 1.1950	< 0.0001
4	− 1.1528	− 1.1658	− 1.1397	< 0.0001	− 1.2258	− 1.2372	− 1.2131	< 0.0001
5	− 1.1356	− 1.1504	− 1.1208	< 0.0001	− 1.2193	− 1.2329	− 1.2057	< 0.0001
6	− 1.1091	− 1.1251	− 1.0932	< 0.0001	− 1.1939	− 1.2085	− 1.1793	< 0.0001
7	− 1.0978	− 1.1155	− 1.0802	< 0.0001	− 1.1834	− 1.1996	− 1.1672	< 0.0001
**Sex, Female (vs. Male)**	− 0.0051	− 0.0114	0.0012	0.1142	− 0.0130	− 0.0193	− 0.0067	< 0.0001
**Age, for 10 years**	− 0.0477	− 0.0499	− 0.0455	< 0.0001	− 0.0365	− 0.0387	− 0.0343	< 0.0001
**Arterial catheter, Yes (vs. No)**	0.2968	0.2889	0.3047	< 0.0001	0.2816	0.2738	0.2893	< 0.0001
**Type of ICU**								
Medical− Surgical ICU	0.0000	0.0000	0.0000		0.0000	0.0000	0.0000	
Cardiac ICU	0.0019	− 0.0068	0.0107	0.6664	− 0.0396	− 0.0487	− 0.0305	< 0.0001
Medical ICU	0.0018	− 0.0092	0.0128	0.7487	− 0.0272	− 0.0377	− 0.0168	< 0.0001
Neuro ICU	− 0.1785	− 0.1933	− 0.1637	< 0.0001	− 0.2013	− 0.2147	− 0.1878	< 0.0001
Surgical ICU	0.0045	− 0.0082	0.0173	0.4837	0.0212	0.0083	0.0341	0.0012
**Apache diagnostic group**								
Cardiac arrest	0.0000	0.0000	0.0000		0.0000	0.0000	0.0000	
Cardiovascular, ACS	− 0.0886	− 0.1054	− 0.0717	< 0.0001	0.0064	− 0.0111	0.0239	0.4713
Cardiovascular, CHF/cardio. shock	0.0515	0.0330	0.0700	< 0.0001	− 0.0076	− 0.0270	0.0119	0.4445
Cardiovascular, other	− 0.0765	− 0.1041	− 0.0489	< 0.0001	0.1079	0.0806	0.1353	< 0.0001
Acute renal failure	0.2047	0.1798	0.2295	< 0.0001	0.0042	− 0.0214	0.0299	0.7463
Respiratory, asthma/emphysema	− 0.0635	− 0.0832	− 0.0439	< 0.0001	− 0.0437	− 0.0628	− 0.0246	< 0.0001
Respiratory, pneumonia	− 0.0769	− 0.0949	− 0.0589	< 0.0001	− 0.0076	− 0.0254	0.0103	0.4065
Respiratory, other	− 0.1076	− 0.1236	− 0.0916	< 0.0001	0.0149	− 0.0017	0.0314	0.0782
Neurology, cerebrovascular accident	− 0.0351	− 0.0528	− 0.0173	0.0001	− 0.0793	− 0.0951	− 0.0635	< 0.0001
Neurology, coma	− 0.0577	− 0.0831	− 0.0323	< 0.0001	− 0.0530	− 0.0767	− 0.0292	< 0.0001
Neurology, other	− 0.1085	− 0.1316	− 0.0853	< 0.0001	− 0.1253	− 0.1448	− 0.1058	< 0.0001
DKA/HHNC	0.7598	0.7423	0.7772	< 0.0001	− 0.1112	− 0.1316	− 0.0908	< 0.0001
Gastrointestinal bleeding	− 0.0782	− 0.0943	− 0.0621	< 0.0001	0.6137	0.5983	0.6292	< 0.0001
Gastrointestinal obstruction	0.0708	0.0413	0.1003	< 0.0001	0.1551	0.1245	0.1857	< 0.0001
Chest pain unknown	− 0.2228	− 0.2604	− 0.1851	< 0.0001	− 0.1336	− 0.1762	− 0.0911	< 0.0001
Overdose	− 0.0831	− 0.1044	− 0.0618	< 0.0001	− 0.1866	− 0.2064	− 0.1668	< 0.0001
Cardiac surgery, valve	0.4235	0.3992	0.4477	< 0.0001	0.4479	0.4222	0.4736	< 0.0001
Cardiac surgery, CABG	0.4835	0.4638	0.5031	< 0.0001	0.4966	0.4757	0.5174	< 0.0001
Sepsis	0.0320	0.0185	0.0455	< 0.0001	0.0505	0.0368	0.0641	< 0.0001
Trauma	0.0329	0.0129	0.0530	0.0013	0.2410	0.2212	0.2608	< 0.0001
Other	− 0.0051	− 0.0195	0.0094	0.4914	0.1011	0.0865	0.1156	< 0.0001
**Apache Score quartile**								
First	0.0000	0.0000	0.0000		0.0000	0.0000	0.0000	
Second	0.1607	0.1508	0.1706	< 0.0001	0.1458	0.1363	0.1553	< 0.0001
Third	0.2933	0.2831	0.3036	< 0.0001	0.2668	0.2568	0.2767	< 0.0001
Fourth	0.4609	0.4502	0.4716	< 0.0001	0.4064	0.3959	0.4170	< 0.0001
**Hospital teaching status, Yes (vs. No)**	0.1836	0.1763	0.1909	< 0.0001	0.1764	0.1691	0.1838	< 0.0001

ACS, Acute coronary syndrome; APACHE, Acute Physiology and Chronic Health Evaluation; CABG, coronary artery bypass grafting CHF/cardio; shock, cardiac heart failure/cardiogenic shock; DKA/HHNC, Diabetic ketoacidosis/hyperglycemic hyperosmolar nonketotic coma; ICU, intensive care unit.

Almost all of the variables were significantly associated with the number of laboratory tests performed per patient per day after adjustment for other variables. The only exceptions were certain APACHE diagnostic groups (for the chemistry and CBC models) and certain types of ICU (for the chemistry model). The number of laboratory tests performed was significantly higher in patients with an arterial catheter (0.2968 [0.2889–0.3047], *P* < 0.0001 for chemistry tests and 0.2816 [0.2738–0.2893], *P* < 0.0001 for CBC tests) or admitted to a teaching hospital (0.1836 [0.1763–0.1909], *P* < 0.0001 for chemistry tests and 0.1764 [0.1691–0.1838], *P* < 0.0001 for CBC tests).

### Factors associated with intensive care unit mortality according to hospital teaching status

An exploratory analysis of the association between ICU mortality and selected variables (sex, age by decade, type of ICU, APACHE diagnostic group, APACHE score) according to hospital teaching status was performed using a logistic regression. The analysis concerned 138,716 patient stays, as 18 patient stays were excluded due to missing data on mortality. The Hosmer–Lemeshow test showed goodness of fit with an alpha risk of 5% (*P* < 0.0001). After adjustment for the other variables, ICU mortality was significantly higher in patient stays in a teaching hospital (adjusted Odds Ratio 1.050 [1.003–1.050], *P* = 0.0369). Table 5 shows the results of this exploratory analysis. Table S3 in supplementary information shows comparison of basic characteristics of patient stays between teaching hospitals and other hospitals.

**Table 5:** Exploratory analysis of the association between intensive care unit mortality and selected variables according to hospital teaching status using logistic regression.

Variable	Adjusted odds ratio	Confidence interval 95%		*p*
**Sex, Male (vs. Female)**	1.043	1.001	1.085	0.0428
**Age, for 10 years**	1.052	1.037	1.067	< 0.0001
**Type of ICU**				
Medical-Surgical ICU	Reference			
Cardiac ICU	1.035	0.980	1.093	0.2162
Medical ICU	1.178	1.104	1.257	< 0.0001
Neuro ICU	1.228	1.121	1.344	< 0.0001
Surgical ICU	1.041	0.953	1.136	0.3719
**Apache diagnostic group**				
Cardiac arrest	Reference			
Cardiovascular, acute coronary syndrome	0.346	0.303	0.396	< 0.0001
Cardiovascular, congestive heart failure/cardiogenic shock	0.537	0.485	0.596	< 0.0001
Cardiovascular, other	0.279	0.233	0.334	< 0.0001
Acute renal failure	0.254	0.213	0.304	< 0.0001
Respiratory, asthma/emphysema	0.362	0.314	0.417	< 0.0001
Respiratory, pneumonia	0.681	0.615	0.754	< 0.0001
Respiratory, other	0.618	0.567	0.673	< 0.0001
Neurology, cerebrovascular accident	0.716	0.651	0.788	< 0.0001
Neurology, coma	0.365	0.311	0.428	< 0.0001
Neurology, other	0.187	0.156	0.224	< 0.0001
Diabetic ketoacidosis/hyperglycemic hyperosmolar nonketotic coma	0.045	0.030	0.066	< 0.0001
Gastrointestinal bleeding	0.293	0.263	0.326	< 0.0001
Gastrointestinal obstruction	0.459	0.378	0.557	< 0.0001
Chest pain unknown	0.198	0.119	0.329	< 0.0001
Overdose	0.057	0.040	0.081	< 0.0001
Cardiac surgery, valve	0.088	0.067	0.114	< 0.0001
Cardiac surgery, coronary artery bypass grafting	0.067	0.054	0.083	< 0.0001
Sepsis	0.581	0.542	0.622	< 0.0001
Trauma	0.439	0.387	0.498	< 0.0001
Other	0.329	0.304	0.355	< 0.0001
**Apache score quartile**				
First	Reference			
Second	2.446	2.162	2.446	< 0.0001
Third	5.541	4.933	5.541	< 0.0001
Fourth	24.622	22.027	24.622	< 0.0001
**Hospital teaching status: Yes (vs. No)**	1.050	1.003	1.050	0.0369

APACHE, acute physiology and chronic health evaluation; ICU, intensive care unit.

## Discussion

This study using a large sample of ICU patients drawn from a recent multicentric database provides a precise and up-to-date description of routine laboratory testing in ICU. The mean number of tests performed per patient on the day of admission was 2.67 for chemistry parameters and 2.42 for CBC parameters. For all parameters, the proportion of patients who underwent at least 1 test decreased from Day-0 to Day-1 and then increased gradually until the end of the ICU stay. Test results evolved towards normal values (*i.e.* tend towards the average of the bounds of the norm) and daily variations in test results decreased over time. An association was found between the use of arterial catheter and the number of laboratory tests performed (0.2968 [0.2889–0.3047], *P* < 0.0001, and 0.2816 [0.2738–0.2893], *P* < 0.0001, respectively for chemistry and CBC tests). The characteristics of ICUs were also associated with the use of laboratory tests, with the number of tests being significantly higher in teaching hospitals than in non-teaching hospitals (0.1836 [0.1763–0.1909], *P* < 0.0001 for chemistry tests and 0.1764 [0.1691–0.1838], *P* < 0.0001 for CBC tests). Mortality in ICU was also found to be significantly higher in teaching hospitals than in non-teaching hospitals (adjusted Odds Ratio 1.050 [1.003–1.050], *P* = 0.0369).

As Karl W. Thomas summarized in a comprehensive editorial, the question of the right test, at the right time, for the right patient, is an old one that has yet to be resolved^2^. However, given the increasingly limited resources available in health care systems across the world, the use of routine laboratory testing in ICU should be optimized urgently. The negative impact of inappropriate laboratory testing has been well demonstrated. Some studies have shown that blood spoliation due to an overuse of laboratory tests can contribute to the onset of anemia and thereby increase the need for blood transfusion in ICU patients^7–9,14–16^. Others believe that the underutilization of laboratory tests may result in delays in management, with lost chance for patients and increased costs of ICU^2^.

Our study using massive, recent, and representative data on “real life” clinical practice can contribute to optimizing the use of routine laboratory testing in ICU. In contrast to earlier studies on the subject, our analysis specifically focused on the relationship between the use of routine laboratory tests and the evolution of laboratory parameters. We found that the proportion of patients who underwent at least 1 test decreased from Day-0 to Day-1 and then increased gradually until the end of the ICU stay for all parameters. Meanwhile, the vast majority of laboratory parameters evolved towards normal values and daily variations in test results decreased over time—with, for example, minimal daily variations in sodium levels of the order of 0.5%. The simultaneous increase in laboratory testing and decrease in parameter variations may seem paradoxical, but it merely reflects the fact that the relationship between routine laboratory testing and the evolution of laboratory parameters is a complex one. A normal test result does not imply that the test was unnecessary. Moreover, a decrease in daily variations can be the result of the close monitoring of laboratory parameters. Lastly, performing a large number of tests despite low daily variations in parameters may be necessary when these tests are aimed at detecting sudden and important variations. To solve this apparent paradox, the real costs of routine laboratory testing should be evaluated by measuring the specific effect of tests on length of stay in ICU, ICU mortality, therapeutic strategies, etc. Large-scale prospective studies should be performed to generate the data needed for this kind of analysis.

Our study showed an association between the use of arterial catheter and the number of laboratory tests. Similarly, the study by Low et al.^17^ found that the number of blood tests and blood-drawing procedures were higher in ICU patients with arterial lines compared to those without. Our data also showed that patient severity (as measured by the APACHE score) increased with the number of tests performed (Table 4). This finding is consistent with the study by Baron et al.^14^, who reported a significant positive correlation between organ dysfunction and the number of blood withdrawals (*r* = 0.34; *P* < 0.001) and total volume withdrawn in critically ill patients (*r* = 0.28; *P* < 0.001).

The number of laboratory tests was associated with hospital teaching status in our study. This finding was to be expected, as several studies have found the volume of laboratory tests to be higher in teaching hospitals than in non-teaching hospitals. Thus, in the 2014 study by Spence et al., the median number of potassium measurements taken during the ICU stay was 4 [3–8] in teaching hospitals vs. 3 [2–6] in non-teaching hospitals^18,19^. The median number of CBC measurements was 4 [3–7] in teaching hospitals and 4 [2–6] in non-teaching hospitals, with an ICU length of stay of 4.0 ± 4.5 and 4.1 ± 4.6 days and APACHE Scores of 14.9 ± 8.0 and 15.6 ± 8.0 for teaching hospitals and non-teaching hospitals, respectively^18^. In addition, our study found, after adjusting for severity, ICU mortality to be higher in teaching hospitals, despite a higher volume of laboratory testing (adjusted Odds Ratio 1.050 [1.003–1.050], *P* = 0.0369). There is no clear explanation for this phenomenon. Both the overuse of laboratory tests and the higher mortality observed in teaching hospitals could be in part attributed to the lack of experience of students. Some authors nevertheless suggest that the lower use of laboratory testing in non-teaching hospitals is proof that the volume of tests can be decreased safely, and that “the price to pay for teaching” could be reduced with corrective measures^2,18^.

This study has some limitations. First, due to the retrospective nature of the study, no causal link between the analyzed variables can be established with certainty. For example, the circumstances that justified the request for a laboratory test and the impact of these laboratory tests on patient management could not be explored in this study. Similarly, we did not analyze separately the laboratory tests measured on point-of-care testing instruments or core laboratory analyzers, which could correspond to examinations done in different clinical approaches. However, our findings can be considered robust since our study draws on a large sample of patients hospitalized in various teaching and non-teaching hospitals across the USA (the eICU-CRD being the most important ICU database in the world). Second, variables not available in the eICU-CRD database could not be analyzed even though they are likely to be associated with the use of laboratory tests in ICU. Indeed, our study would have been stronger if the impact of prescriber experience on the number of tests performed had been included in the analysis. Likewise, it would have been interesting to assess the impact of local protocols for the prescription of laboratory tests, especially since such protocols have shown their usefulness in reducing the number of tests without changing the prognosis of patients^5,6,20^. Future studies should explore the link between these variables and routine laboratory testing once the necessary data become available. These results, with stable and often normal results, are in favor of the application of a method of prediction of results in order to provide assistance in the prediction of biological medical examinations.

## Conclusions

This retrospective, descriptive multi-center study using a very large sample of ICU patients showed that the number of routine laboratory tests performed during the first 8 days of ICU stay is very high. Another key finding is that the proportion of patients tested daily increases over time, even as the vast majority of laboratory parameters evolve towards normal values. This paradox should be further explored by assessing the factors that drive the decision to perform routine laboratory tests in ICU as well as the impact of these tests on patient management and outcome.

## Supplementary Information

Supplementary Tables.

## Data Availability

The data used for this study are from the eICU-CRD database. This database is publicly available after registration. The data are fully available on request from the database managers: https://eicu-crd.mit.edu/.

## Abbreviations

ICU: Intensive care unit
CBC: Complete blood count
eICU-CRD: EICU Collaborative Research Database
APACHE: Acute Physiology and Chronic Health Evaluation
WBC: White blood count
RBC: Red blood count
IQR: Inter-Quartile Range
GEE: Generalized estimating equation
