# Supplementary material for: A descriptive study of routine laboratory testing in intensive care unit in nearly 140,000 patient stays
Source: Sci Rep. 2022 Dec 13;12:21526. doi: 10.1038/s41598-022-25961-1 (PMC9747911; doi:10.1038/s41598-022-25961-1)
Supplement: Supplementary file 1 — Supplementary Tables. [file 41598_2022_25961_MOESM1_ESM.docx]

Table S1 : Comparison of included patient stays with excluded patient stays due to APACHE score prediction error.

| Variable | Included  (n = 138,734) | Excluded  (n = 54,335) | Standardized Difference |
| --- | --- | --- | --- |
| Age, mean (SD) | 62.2 (16.5) | 62.3 (16.6) | 0.01 |
| Gender, male, n (%) | 75,858 (54.7%) | 29,419 (54.1%) | -0.01 |
| Teaching hospital, n (%) | 38,649 (27.9%) | 11,429 (21.0%) | -0.16 |
| Catecholamine, n (%) | 20,547 (14.8%) | 3,427 (6.3%) | -0.28 |
| Arterial catheter, n (%) | 38,028 (27.4%) | 7,639 (14.1%) | -0.33 |
| Mechanical ventilation, n (%) | 34,152 (24.6%) | 7,030 (12.9%) | -0.30 |
| Number of chemistry per day, median (Q1-Q3) | 1.0 (1.0 – 2.0) | 1.0 (0.0 – 2.0) | -0.25 |
| Number of CBC per day, median (Q1-Q3) | 1.0 (1.0 – 2.0) | 1.0 (0.0 – 1.0) | -0.26 |
| ICU Length of Stay, day, median (Q1-Q3) | 2.0 (1.0 – 4.0) | 1.0 (1.0 – 2.0) | -0.23 |
| Hospital Length of Stay, day, median (Q1-Q3) | 5.0 (3.0 – 9.0) | 4.0 (2.0 – 8.0) | -0.05 |

APACHE score : Acute Physiology and Chronic Health Evaluation Score, CBC: Complete blood count, ICU: Intensive Care Unit, median (Q1 – Q3): median (first quartile – third quartile), SD: Standard deviation.

Table S2: Number and percentage of laboratory results considered as outliers

| Variable | Total before outliers exclusion (n) | Outliers excluded (n) | Outliers excluded (%) |
| --- | --- | --- | --- |
| Potassium | 1,367,657 | 2,214 | 0.162 |
| Sodium | 1,275,499 | 2,370 | 0.186 |
| Chloride | 1,188,472 | 2,309 | 0.194 |
| Bicarbonate | 1,111,638 | 1,750 | 0.157 |
| Creatinine | 1,182,032 | 2,219 | 0.188 |
| Urea | 1,176,398 | 1,640 | 0.139 |
| White blood count | 1,053,744 | 1,859 | 0.176 |
| Red blood count | 1,050,494 | 2,067 | 0.197 |
| Platelet count | 1,056,585 | 2,079 | 0.197 |
| Hemoglobin | 1,190,249 | 2,309 | 0.194 |

Table S3: Comparison of basic characteristics of patient stays between teaching hospitals and other hospitals.

| Variable | Teaching Hospital  (n = 38,649) | Other Hospital  (n = 100,085) | Standardized Difference |
| --- | --- | --- | --- |
| Age, mean (SD) | 60.9 (16.5) | 62.7 (16.4) | -0.11 |
| Gender, male, n (%) | 21,429 (55.5%) | 54,429 (54.4%) | 0.02 |
| APACHE Score, mean (SD) | 56.1 (26.3) | 54.6 (25.2) | 0.06 |
| Catecholamine, n (%) | 6,033 (15.6%) | 14,514 (14.5%) | 0.03 |
| Arterial catheter, n (%) | 13,858 (35.9%) | 24,170 (24.2%) | 0.26 |
| Mechanical ventilation, n (%) | 10,196 (26.4%) | 23,956 (23.9%) | 0.06 |
| Number of chemistry per day, median (Q1-Q3) | 1.0 (1.0 – 2.0) | 1.0 (1.0 – 2.0) | 0.18 |
| Number of CBC per day, median (Q1-Q3) | 1.0 (1.0 – 2.0) | 1.0 (1.0 – 2.0) | 0.16 |
| ICU Length of Stay, day, median (Q1-Q3) | 4.0 (3.0 – 7.0) | 4.0 (2.0 – 7.0) | 0.15 |
| Hospital Length of Stay, day, median (Q1-Q3) | 8.0 (5.0 – 14.0) | 7.0 (4.0 – 12.0) | 0.16 |

APACHE score : Acute Physiology and Chronic Health Evaluation Score, CBC: Complete blood count, ICU: Intensive Care Unit, median (Q1 – Q3): median (first quartile – third quartile), SD: Standard deviation.
